# A284 EVALUATING THE INCIDENCE OF MAJOR GASTROINTESTINAL BLEEDING IN PATIENTS WITH CIRRHOSIS ON ANTICOAGULATION THERAPY: A TERTIARY SINGLE CENTRE EXPERIENCE

**DOI:** 10.1093/jcag/gwad061.284

**Published:** 2024-02-14

**Authors:** H Bualbanat, A Teriaky, D Hudson, K Qumosani

**Affiliations:** Gastroenterology, Western University, London, ON, Canada; Gastroenterology, Western University, London, ON, Canada; Gastroenterology, Western University, London, ON, Canada; Gastroenterology, Western University, London, ON, Canada

## Abstract

**Background:**

Patients with chronic liver disease, especially those with cirrhosis, face an increased risk of bleeding and thrombosis due to hemostasis dysregulation. In such cases, prescribing anticoagulation therapy requires a cautious approach, given the inherent risk-reduction in thrombosis alongside a suspected elevated bleeding risk.

**Aims:**

Perform a retrospective study to evaluate anticoagulation safety and gastrointestinal bleeding incidence in cirrhotic patients.

**Methods:**

A retrospective study was conducted at the University Hospital of London Health Sciences Centre, involving patients who attended outpatient hepatology clinics between 2010 and 2022. To be eligible, patients had to be 18 years or older and have confirmed cirrhosis through imaging, radiology, or pathology. They were monitored for up to 5 years for the primary outcome, which was major gastrointestinal bleeding requiring hospitalization. Exclusions included patients with underlying hematologic or thrombotic conditions contributing to abnormal hemostasis beyond cirrhosis and those who underwent liver transplantation. Descriptive statistics were provided, such as means and standard deviations for continuous variables, and proportions/percentages for categorical ones. Group comparisons utilized the Student's t-test for continuous variables and chi-squared tests for categorical variables. Univariate and multivariate logistic regression were employed to calculate odds ratios and their associated 95% confidence intervals for the primary outcome

**Results:**

We retrospectively enrolled 300 patients, with 34.0% having cirrhosis and anticoagulation history. The average age was 63.1 years [95% CI 61.7 – 64.6], and 43.7% were female. Atrial fibrillation was the primary reason for anticoagulation (53.9%). Cirrhotic patients on anticoagulation had a 13.1% bleeding rate, while those without had 18.6% (p-value = 0.207, not significant). Univariate analysis suggested a possible link between anticoagulation and bleeding risk (OR 1.51 [95% CI 0.79 – 2.89]). No covariates predicted bleeding in univariate logistic regression. In multivariate analysis adjusting for age and sex, the odds ratio remained at 1.50 [95% CI 0.78 – 2.88].

The study hints at a higher bleeding risk in cirrhotic patients on anticoagulation, but more statistical power is needed, either through a larger sample or a multicenter approach.

**Conclusions:**

To further investigate the association between anticoagulation and bleeding risk in cirrhotic patients, a larger multicentre study with increased enrollment is required. Nonetheless, interim findings do suggest the potential existence of an associated bleeding risk in cirrhotic patients undergoing anticoagulation therapy, albeit with a small observed effect size and uncertain clinical significance.

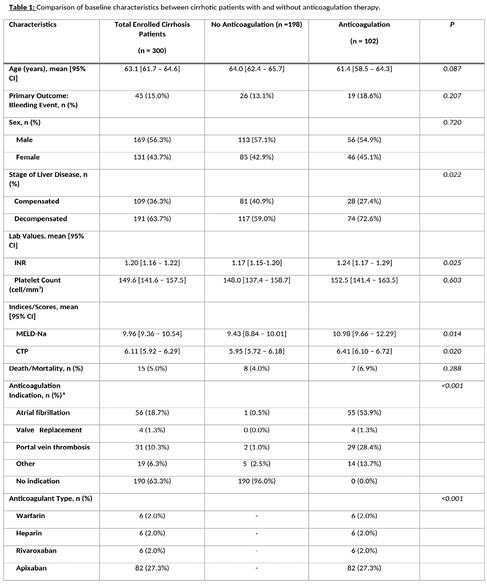

**Funding Agencies:**

None

